# Preoperative prediction of perineural invasion with multi-modality radiomics in rectal cancer

**DOI:** 10.1038/s41598-021-88831-2

**Published:** 2021-05-03

**Authors:** Yu Guo, Quan Wang, Yan Guo, Yiying Zhang, Yu Fu, Huimao Zhang

**Affiliations:** 1 Department of Radiology, The First Hospital of Jilin University, Jilin Provincial Key Laboratory of Medical Imaging and Big Data, Changchun, China; 2 Department of Gastric and Colorectal Surgery, The First Hospital of Jilin University, Changchun, China; 3 GE Healthcare, Shanghai, China

**Keywords:** Cancer imaging, Bioinformatics, Predictive markers, Rectal cancer

## Abstract

Perineural invasion (PNI) as a grossly underreported independent risk predictor in rectal cancer is hard to identify preoperatively. We aim to predict PNI status in rectal cancer using multi-modality radiomics. In total, 396 radiomics features were extracted from T2-weighted images (T2WIs), diffusion-weighted images (DWIs), and portal venous phase of contrast-enhanced CT (CE-CT) respectively of 94 consecutive patients with histologically confirmed rectal cancer. T2WI score, DWI score, and CT score were calculated via the radiomics features selection and optimization. Discrimination, calibration, and clinical benefit ability were used to evaluate the performance of the radiomics scores in both training and testing datasets. CT score and T2WI score were independent risk predictors [CT score, OR (95% CI) = 4.218 (1.070–16.620); T2WI score, OR (95% CI) = 105.721 (3.091–3615.790)]. The concise score which combined CT score and T2WI score, showed the best performance [training dataset, AUC (95% CI) = 0.906 (0.833–0.979); testing dataset, AUC (95% CI) = 0.884 (0.761–1.000)] and good calibration (P > 0.05 in the Hosmer–Lemeshow test for the training and testing datasets). Decision curve analysis showed that the multi-modality radiomics nomogram had a higher clinical net benefit. The multi-modality radiomics score could be used to preoperatively assess PNI status in rectal cancer.

## Introduction

Rectal cancer is one of the most common cancers and the leading causes of death around the world^1,2^. Perineural invasion (PNI), malignant invasion of nervous structures and nerve sheaths that could be a source of metastatic spread beyond the extend of any local invasion, indicating an increased risk for poor prognosis and decreased survival in rectal cancer^3–6^.

Although the molecular mechanisms behind PNI are obscure, nerves are also regarded as another route of malignant cells dissociate from the local primary tumor to deposit metastases except for vascular and lymphatic channels^3^. PNI is a grossly underreported predictor in rectal cancer, the National Comprehensive Cancer Network describes PNI as an important parameter to be reported in the standard pathological reports^7^. Total mesorectum excision (TME) is the main therapy choice for rectal cancer, and recent studies proved that PNI-positive rectal cancer proves to experience incomplete tumor resection and local tumor recurrence^5,8^. Liebig et al. reported that about 30–40% of rectal cancer are PNI-positive rectal cancer who with a 5-years overall survival of less than 16% and median survival time (MST) of 25 months after surgical resection^8^. Some studies proved that PNI-positive patients could get potential benefits from adjuvant therapy and PNI status should be considered when stratifying rectal cancer patients for adjuvant treatment. Hence, there is a significant clinical implication to detect PNI status preoperatively^9,10^.

The identification of the PNI status preoperatively is based on biopsy specimens which were reviewed by pathologists in photomicrographs. Efficiency and timeliness of detecting PNI status can limit the application to assist cancer treatment decisions^11–13^. Magnetic resonance imaging (MRI) and computer tomography (CT) play a vital role in the rectal tumor detecting, location and the extent of disease defining, treatment planning, and longitudinal response monitoring, which are noninvasively compared with other procedures such as preoperative biopsy and serum test^14–16^. Nevertheless, the traditional radiological image fails to detect the PNI status preoperatively.

Combined with artificial intelligence (AI), MRI and CT would be used to assess tumor phenotype and its local microenvironment which could provide additional information to determining potential treatments in the advanced image analysis algorithms^17^. Currently, this kind of AI-based process to analyze medical imaging and provide more quantitative image features is referred to as "radiomics"^18–20^. In recent years, radiomics have shown effective diagnostic, prognostic, and predictive ability in the clinical decision support systems of oncology^14,21–26^. As important components of the tumor microenvironment, Huang et al. reported that radiomics-based CT model status could predict PNI status in colorectal cancer^27^. However, most of radiomics analyses in oncology usually depend on the unimodality equipment such as CT, MRI, or ultrasound, et cetera^19,20^. Multi-modal machine learning (MMML) is a process that could be used to combine information from multiple modalities and perform a target prediction^28,29^. However, there is still a rare of multi-modality radiomics-based methods to predict PNI status for rectal cancer.

Thus, we aim to investigate the potential value of multi-modality radiomics derived from MRI and CE-CT for the preoperative prediction of PNI status in rectal cancer patients.

## Results

### Patient characteristics

A total of 94 patients were included. For stage I-II rectal cancer, 40 (88.9%) patients performed radical surgical treatment. 5 (11.1%) patients performed surgery followed by chemotherapy; For stage III rectal cancer, 22 (44.9%) patients performed radical surgical treatment. 24 (49.0%) patients performed surgery followed by chemotherapy. 2 (4.1%) patients performed surgery followed by radiotherapy. 1 (2.0%) patient performed surgery followed by chemoradiotherapy. Included patients were divided into the training dataset (21 PNI-positive rectal cancer and 44 PNI-negative rectal cancer) and testing dataset (10 PNI-positive rectal cancer and 19 PNI-negative rectal cancer) randomly. There was no significant difference between the difference of clinical characteristics between the training dataset and testing dataset (P = 0.099–1.000) (Table 1). In the univariable logistic regression analysis, the initially selected clinical risk predictors included CA19-9, T staging, and N staging diagnosed by pathology (all P < 0.05). After the multivariable logistic regression analysis, N staging was regarded as independent risk predictors to PNI status (P < 0.05) (Table 2). There was more T3-4 rectal cancer (97.09% vs 55.55%) and N1-2 rectal cancer (80.65% vs 38.09%) in the PNI-positive group than in the PNI-negative group (both P < 0.05). However, the radiomics model was established preoperatively, N staging wouldn’t be contained in the radiomics model.

**Table 1:** Clinical characteristics and radiomics scores for the perineural invasion in the training and testing datasets.

Variables	All patients (N = 94)	Training dataset (N = 65)	Testing dataset (N = 29)	*P* value
**PNI status**				1
Negative	63 (67.02)	44 (67.69)	19 (65.52)	
Positive	31 (32.98)	21 (32.31)	10 (34.48)	
Age	59.20 ± 12.43	59.37 ± 12.94	58.83 ± 11.41	0.846
**Gender**				0.707
Male	72 (76.60)	51 (78.46)	21 (72.41)	
Female	22 (23.40)	14 (21.54)	8 (27.59)	
**CEA level**				0.757
≤ 3.4 ng/ml	46 (48.94)	33 (50.77)	13 (44.83)	
> 3.4 ng/ml	48 (51.06)	32 (49.23)	16 (55.17)	
**CA19-9 level**				1
≤ 22 ng/ml	78 (82.98)	54 (83.08)	24 (82.76)	
> 22 ng/ml	16 (17.02)	11 (16.92)	5 (17.24)	
**Tumor location**				0.916
Middle and high	51 (54.26)	36 (55.38)	15 (51.72)	
Low	43 (45.74)	29 (44.62)	14 (48.28)	
Tumor length (cm)	5.25 (4.10, 6.78)	5.60 (4.60, 6.70)	4.70 (3.40, 7.10)	0.271
Tumor thickness(cm)	1.40 (1.10, 1.60)	1.40 (1.10, 1.60)	1.40 (1.10, 1.70)	0.908
**R_T staging**				0.466
T 1–2	23 (24.47)	14 (21.54)	9 (31.03)	
T 3–4	71 (75.53)	51 (78.46)	20 (68.97)	
**R_N staging**				1
N0	25 (26.60)	17 (26.15)	8 (27.59)	
N1–2	69 (73.40)	48 (73.85)	21 (72.41)	
**P_T staging**				0.443
T 1–2	32 (34.04)	20 (30.77)	12 (41.38)	
T 3–4	62 (65.96)	45 (69.23)	17 (58.62)	
**R_N staging**				0.864
N0	45 (47.87)	32 (49.23)	13 (44.83)	
N1–2	49 (52.13)	33 (50.77)	16 (55.17)	
T2WI score	− 0.79 (− 1.10, − 0.45)	− 0.77 (− 1.09, − 0.39)	− 0.80 (− 1.18, − 0.53)	0.697
DWI score	− 0.69 (− 0.78, − 0.59)	− 0.69 (− 0.78, − 0.60)	− 0.69 (− 0.76, − 0.59)	0.990
CT score	− 0.90 (− 1.57, − 0.41)	− 0.79 (− 1.52, − 0.40)	− 1.14 (− 1.93, − 0.76)	0.099

Data are presented as mean ± SD, No. (%) or median (25%, 75%). *PNI* perineural invasion, *CEA* carcinoembryonic antigen, *CA19-9* cancer antigen 19–9, *R_T staging* T staging was diagnosed based on rectal MR and CT images, *R_N staging* N staging was diagnosed based on rectal MR and CT images, *P_T staging* T staging was diagnosed by pathology, *P_N staging* N staging was diagnosed by pathology.

**Table 2:** Univariate and multivariable logistic regression analysis for the prediction of PNI status.

Characteristics	Univariate analysis			Multivariate analysis		
	β	Odds ratio (95% CI lower–upper)	P value	β	Odds ratio (95% CI lower–upper)	P value
Age	− 0.019	0.982 (0.943–1.022)	0.369	–	–	–
Gender	0.693	2.000 (0.493–8.109)	0.332	–	–	–
CEA (> 3.4 ng/ml)	0.968	2.632 (0.888–7.794)	0.081	–	–	–
CA19-9 (> 22 ng/ml)	− 1.609	0.200 (0.051–0.788)	0.021	− 2.133	0.118 (0.009–1.514)	0.101
Tumor location	0.395	1.484 (0.514–4.287)	0.466	–	–	–
Tumor length	0.062	1.064 (0.817–1.386)	0.643	–	–	–
Tumor thickness	0.607	1.834 (0.768–4.382)	0.172	–	–	–
R_T staging	1.270	3.562 (0.719–17.662)	0.120	–	–	–
R_N staging	1.030	2.800 (0.706–11.097)	0.143	–	–	–
P_T staging	− 1.884	0.152 (0.031–0.735)	0.019	− 0.151	0.860 (0.086–8.606)	0.898
P_N staging	− 2.007	0.134 (0.039–0.469)	0.002	− 2.404	0.09 (0.009–0.950)	0.045
T2WI score	2.662	14.331 (2.955–69.488)	0.001	4.661	105.721 (3.091–3615.790)	0.010
DWI score	4.294	73.234 (0.841–6374.624)	0.060	0.454	1.575 (0.001–2063.812)	0.901
CT score	1.889	6.615 (2.182–20.055)	0.001	1.439	4.218 (1.070–16.620)	0.040

*β* regression coefficient, *95% CI* lower–upper, the lower value to the upper value of 95% confidence interval, *CEA* carcinoembryonic antigen, *CA19-9* cancer antigen 19–9, *R_T staging* T staging was diagnosed based on rectal MR and CT images, *R_N staging* N staging was diagnosed based on rectal MR and CT images, *P_T staging* T staging was diagnosed by pathology, *P_N staging* N staging was diagnosed by pathology.

### Radiomics scores

128 radiomics features in the modality of T2WI, 181 radiomics features in the modality of DWI, 65 radiomics features in the modality of portal venous phase CE-CT, which showed stability with good inter-/intra observer agreement (ICCs > 0.70) had been remained. The radiomics features extracted from reader 1’s segmentation with stable inter-/intra-observer reproducibility went through the selection process.

In the process of building radiomics score in the modality of T2WI, DWI, and portal venous phase CE-CT, the minimum redundancy maximum correlation (mRMR) selection was used to select the most relevant and the least redundant 20 radiomics features to PNI status respectively. Then the least absolute shrinkage and selection operator (LASSO) method was used. The lambdas were chosen as 0.051, 0.095, and 0.004 for the T2WI radiomics features, DWI radiomics features, and portal venous phase CE-CT radiomics features, respectively and which gave the minimum binominal deviance. 5 T2WI radiomics features (Supplementary Information 1: Figure S1), 1 DWI radiomics feature (Supplementary Information 1: Figure S2), and 12 portal venous phase CE-CT radiomics features (Supplementary Information 1: Figure S3) remained. The radiomics scores were calculated by the selected features multiplied the corresponding coefficients for each modality as follows:

$$ \begin{gathered} T2WI\;{\text{score }} = \, - 0.761 \, + { (} - \, 0.258 \, \times {\text{LongRunEmphasis}}\_{\text{angle}}0\_{\text{offset}}4 \, - 0.131 \times {\text{DifferenceVariance }} - \, 0.102 \times {\text{LongRunEmphasis}}\_{\text{angle}}45\_{\text{offset}}4 \, - 0.087 \hfill \\ \times {\text{RunLengthNonuniformity}}\_{\text{AllDirection}}\_{\text{offset}}4\_{\text{SD }} + 0.408 \times {\text{MaxIntensity)}} \hfill \\ \end{gathered} $$

$$ {\text{DWI}}\;{\text{score }} = \, - 0.719 \, - \, 0.144 \times {\text{InverseDifferenceMoment}}\_{\text{angle}}0\_{\text{offset}}1 $$

$$ \begin{gathered} {\text{CT}}\;{\text{score }} = \, - 0.988 \, + \, ( - 0.436 \times {\text{Sphericity }} + 2.838 \times {\text{Quantile}}\;0.975 \, + 0.857 \times {\text{LongRunLowGreyLevelEmphasis}}\_{\text{angle}}45\_{\text{offset}}4 \, + 0.874 \times {\text{Percentile}}15 \, - 0.665 \times {\text{ShortRunLowGreyLevelEmphasis}}\_{\text{AllDirection}}\_{\text{offset}}7\_{\text{SD }} + 1.25 \times {\text{HaralickCorrelation}}\_{\text{AllDirection}}\_{\text{offset}}4\_{\text{SD}} \hfill \\ - 0.511 \times {\text{GLCMEnergy}}\_{\text{angle}}135\_{\text{offset}}7 \, + 0.828 \times {\text{ClusterShade}}\_{\text{AllDirection}}\_{\text{offset}}1\_{\text{SD }} + 2.131 \times {\text{LowGreyLevelRunEmphasis}}\_{\text{angle}}90\_{\text{offset}}1 \, - 3.153 \times {\text{Percentile}}90 \, - 2.377 \times {\text{GLCMEnergy}}\_{\text{angle}}45\_{\text{offset}}7 \, - 2.067 \times {\text{ShortRunLowGreyLevelEmphasis}}\_{\text{angle}}0\_{\text{offset}}1) \hfill \\ \end{gathered} $$

T2WI score and CT score showed a significant difference between the PNI-positive group and the PNI-negative group in the training and testing datasets (all P < 0.05) (Table 1). In the univariable logistic regression analysis, the initially selected radiomics risk predictors included T2WI score and CT score (all P < 0.05). After the multivariable logistic regression analysis, the T2WI score and CT score were regarded as independent risk predictors to PNI status (all P < 0.05) (Table 2). T2WI score and DWI score were combined as MR score. T2WI score and CT score were combined as concise score. T2WI score, DWI score, and CT score were combined as integrated score. The multi-modality radiomics scores were combined by the single modality radiomics score multiplied the corresponding coefficients as follows:

$$ {\text{MR}}\;{\text{score}} = 2.921 + 2.551*{\text{T2WI}}\;{\text{score}} + 2.756*{\text{DWI}}\;{\text{score}} $$

$$ {\text{Concise}}\;{\text{score}} = 2.078 + 2.400*{\text{T2WI}}\;{\text{score}} + 1.842*{\text{CT}}\;{\text{score}} $$

$$ {\text{Integrated}}\;{\text{score}} = 1.898 + 2.422*{\text{T2WI}}\;{\text{score}} - 0.298*{\text{DWI}}\;{\text{score}} + 1.856*{\text{CT}}\;{\text{score}} $$

### Performance of the radiomics scores

**Figure 1:**
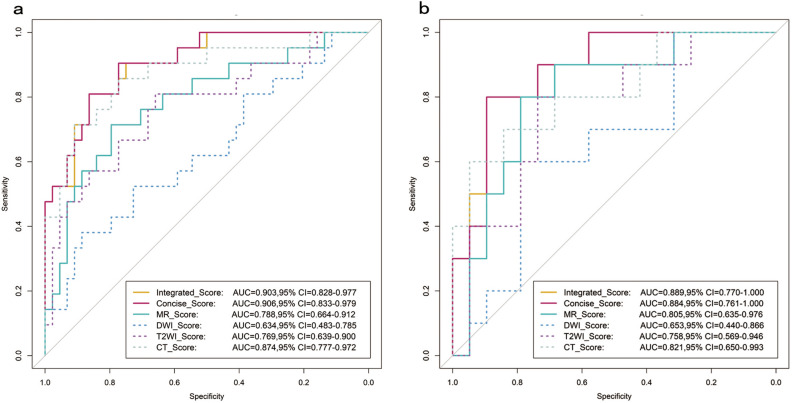
Receiver operating characteristic curves analysis in training and testing datasets. Receiver operating characteristic curves analysis in training (**a**) and testing datasets (**b**) for each radiomics models to predict perineural invasion status; *AUC* area under the curve, *95% CI* 95% confidence interval.

Among the single modality models, the CT score showed the best discrimination. The area under the curve (AUC) of CT score was 0.874 (95% CI 0.777–0.972) in the training dataset and 0.821 (95% CI 0.650–0.993) in the testing dataset. The discrimination performance was better than the DWI score in the training and testing datasets (both P < 0.05) (Fig. 1). The best discrimination performance of derived multi-modality radiomics models was the concise score which combined the T2WI score and the CT score. The AUC of the concise score was 0.906 (95% CI 0.833–0.979) in the training dataset and 0.884 (95% CI 0.761–1.000) in the testing dataset. The concise score was higher in the PNI-positive group than the PNI-negative group in both training and testing datasets (Supplementary Information 1: Figure S4). AUC, specificity, sensitivity, and accuracy of every single modality radiomics score and derived radiomics scores were showed in Table 3, in which the Delong test was used to verify the radiomics scores were robust both in the training and testing datasets.

**Table 3:** The discrimination performance of models in the training and testing datasets.

Model	Cutoff	Training dataset				Testing dataset				P value
		AUC (95% CI lower–upper)	SPE	SEN	ACC	AUC (95% CI lower–upper)	SPE	SEN	ACC	
T2WI score	− 0.768	0.769 (0.639–0.900)	0.659	0.810	0.708	0.758 (0.569–0.946)	0.759	0.800	0.759	0.922
DWI score	− 0.578	0.634 (0.483–0.785)	0.886	0.789	0.723	0.653 (0.440–0.866)	0.789	0.200	0.586	0.890
CT score	− 0.713	0.874 (0.777–0.972)	0.773	0.857	0.800	0.821 (0.650–0.993)	0.947	0.600	0.828	0.598
MR score	− 0.555	0.788 (0.664–0.912)	0.795	0.714	0.769	0.805 (0.635–0.976)	0.789	0.600	0.724	0.872
Integrated score	− 0.656	0.903 (0.828–0.977)	0.864	0.810	0.846	0.889 (0.770–1.000)	0.895	0.500	0.759	0.856
Concise score	− 1.254	0.906 (0.833–0.979)	0.773	0.905	0.815	0.884 (0.761–1.000)	0.895	0.600	0.793	0.768

When the model value lesser than the corresponding cut-off value means perineural invasion status is negative; *AUC* the area under receiver operating characteristic (ROC) curves, *95% CI lower–upper* the lower value to the upper value of 95% confidence interval, *SPE* specificity, *SEN* sensitivity, *ACC* accuracy; P value was derived from Delong test between the training and testing datasets.

**Figure 2:**
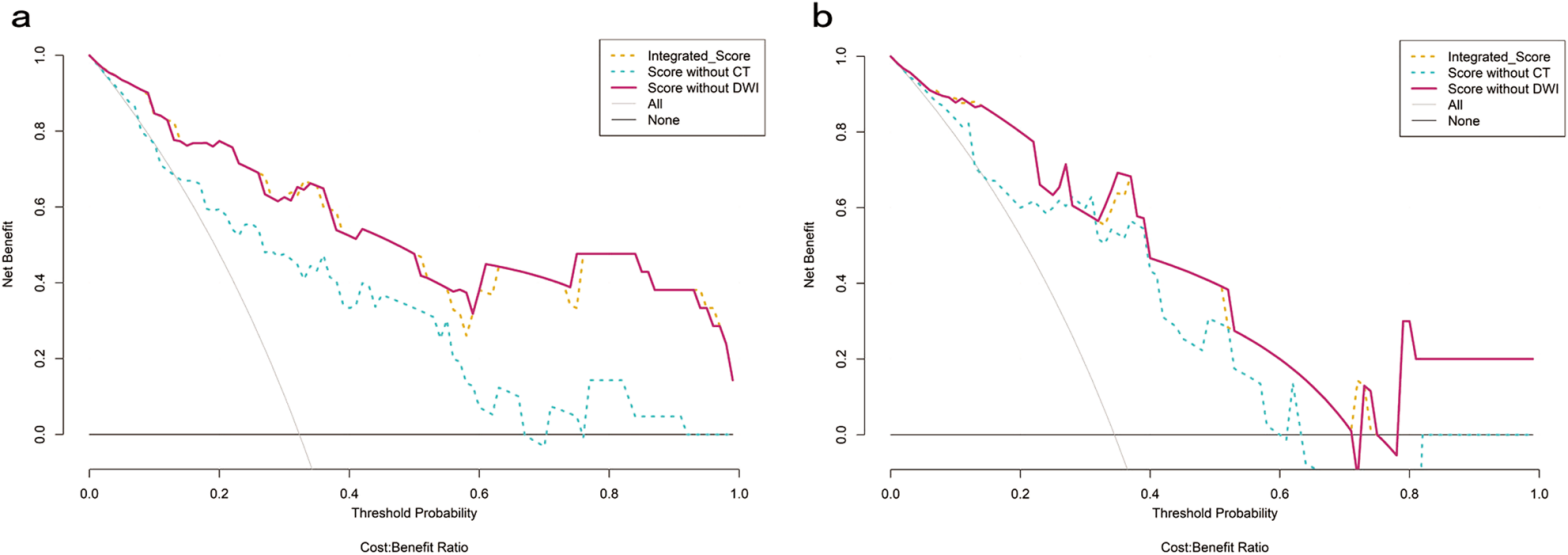
Decision curve analysis of radiomics models for predicting perineural invasion status. (**a**) Training dataset; (**b**) Testing dataset. The y-axis measures the probability of net benefit. The x-axis measures the threshold probability of perineural invasion positive. The red solid curve represents concise score. The blue and yellow dotted curve represents MR score and integrated score, respectively. The grey curve represents the assumption that all perineural invasion status is positive. The black line represents the assumption that all perineural invasion status is negative.

**Figure 3:**
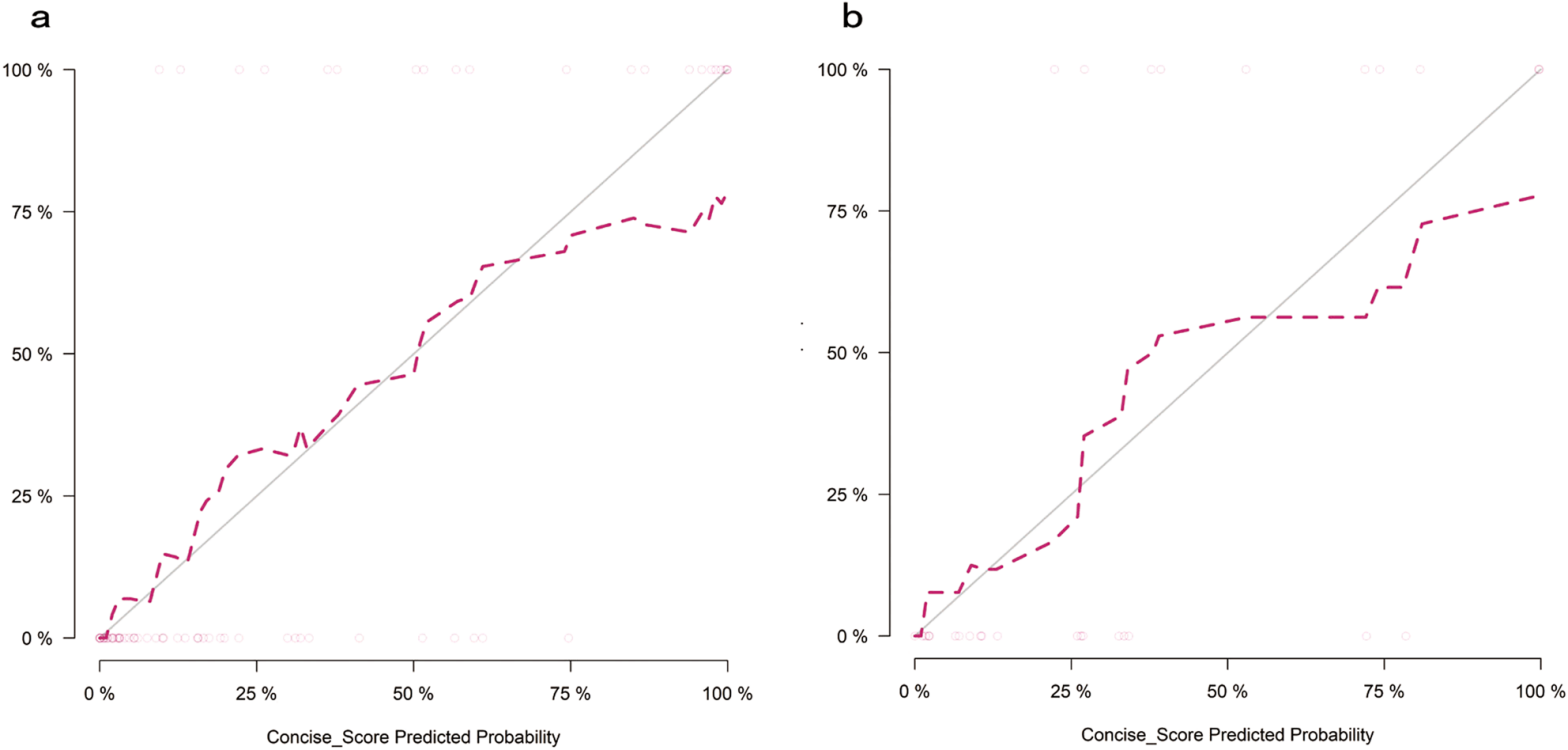
Calibration curves of the radiomics nomogram in the training and testing datasets. (**a**) Training dataset; (**b**) Testing dataset. P > 0.05 in the Hosmer–Lemeshow test for the training and testing datasets, which indicating an appropriate agreement between the predicted perineural invasion status and actual observed perineural invasion status. The y-axis represents the actual perineural invasion probability. The x-axis represents the predicted perineural invasion probability. The grey solid line represents the best model with the perfect agreement. The red dotted curves have a closer fit to the grey solid line represents the radiomics nomogram has a better calibration.

**Figure 4:**
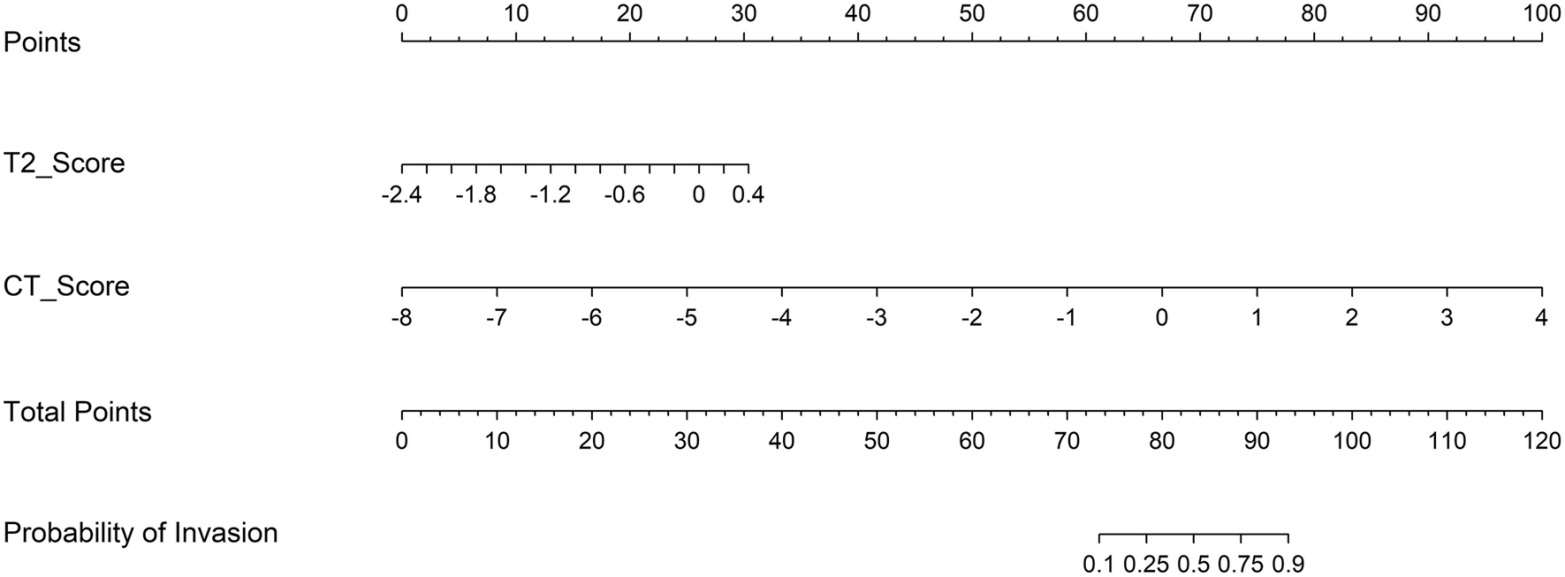
Radiomic nomogram was presented to predict Perineural Invasion status in rectal cancer. The nomogram was built in the training dataset with the T2 score and CT score using a multivariate logistic regression model. The range of T2 score and CT score are respectively − 2.4 to 0.4 and − 8 to 4. The proportional regression coefficients of T2 score and CT score were scaled in the points. Sum the points of T2 score and CT score to get the total points in points scale. The probability of perineural invasion in a rectal cancer patient is the corresponding numbers on the lower probability of Invasion scale.

In most sections of the DCA curves, the concise score is at the top right comparing with the MR score. The concise score and the integrated score have a comparable net benefit, which show several overlaps within the full range of threshold probabilities. The concise score and the integrated score have similar clinical application value, both of which are higher than MR score (Fig. 2). The concise score was appointed as the final model, which had a better discrimination ability and clinical benefit. The calibration curve of the concise score showed good agreement between predicted probability value and the real value in both training and testing datasets (P > 0.05) (Fig. 3). In order to ensure the model to be used easily, the concise score consisting of two radiomics scores was presented as the nomogram (Fig. 4)**.**

## Discussion

An initial objective of our study was to develop and validate a multi-modality radiomics model for preoperative prediction of PNI status in rectal cancer and the multi-modality radiomics model was converted into a nomogram which is easy to be used in the individual cancer treatment decision system. In the results, the concise score (multi-modality radiomics model integrating T2WI score and CT score) showed excellent discrimination and calibration ability. Nevertheless, the DWI score failed to provide additional predictive value to assess PNI status in rectal cancer.

Among the clinical characteristics, there was no statistically significant difference between the training dataset and the testing dataset. As for the clinical candidate predictor, CA19-9 showed a statistical difference between abnormal tumor marker-level and PNI status in the univariate logistic regression analysis, which is consistent with the published study^30^. Some studies showed the statistical difference between normal level and abnormal levels of CA19-9 and CEA in the PNI-positive and PNI-negative rectal cancer^6,31^. Whereas, there is no sufficient evidence to prove tumor markers could be regarded as an independent risk predictor for PNI status in rectal cancer. It is argued that nonsignificant statistical association risk factor should be kept in the model development. However, with a small sample size, caution must be applied in the candidate factor selection avoiding overfitting. Finally, it is failed to be absorbed in the multi-modality radiomics model. As for the histopathological predictors, N staging diagnosed by pathology is an independent risk predictor for PNI status in rectal cancer in the multivariate logistic regression analysis. Following the present results, previous studies have demonstrated a correlation between PNI status and advanced tumor stage^5,6^. However, the integration of histopathological predictors from surgical tissue may cause sampling bias and be tend to high false-negative rate. On the other hand, the multi-modality radiomics model was established before the surgery, the relevant postoperative pathological characteristics were not involved in our model. Our results confirmed that radiomics can use noninvasive CT and MR scans performed in the clinical diagnosis procedure to predict the PNI status in rectal cancer^20,32^, which is corroborate the previous work that taking different aspects marker is the most promising way to facilitates the individualized clinical management^33^. This main finding is based on the central concept that biomedical images reflect underlying pathophysiology via mineable high-dimensional and quantitative image analysis, such as tumor microenvironment. PNI status is regarding as an important component of the tumor microenvironment^17–19^. To construct the radiomics score for each imaging modality of VOIs, an integrated radiomics selection was performed. The inter- and intra-class ICCs were used to select the stable radiomics features. This method could avoid the feature fluctuations caused by segmentation and accomplish the robustness of model development. The mRMR selection and LASSO algorithm were applied to reduce superfluous redundancy radiomics features. This combined method could choose the most relevant radiomics features and eliminate the most superfluous radiomics features to the PNI status. Completing the process of fivefold cross-validation to ensure the stability of the selected radiomics features and summing a panel of selected radiomics features weighted by the regression coefficients to construct the radiomics score. In the formula of our radiomics scores, the Energy of the Grey level Co-occurrence Matrix (GLCM) were selected regarding as a negative relationship with PNI-positive rectal cancer. It not only supports the published research^27^ but also verifies the theory that higher the heterogeneity of tumor represents stronger the invasiveness^34,35^. Comparing with the previous research, besides first-order statistics features and high order statistics features, morphological features were also be extracted from the VOIs of the rectal tumor. Sphericity was chosen as a negative element and a possible explanation for this might be that rectal cancer with the shape of the round may tend to be less aggressive. Most of the selected radiomics features in the modality of T2WI belonged to Gray Level Run-Length Matrix (GLRM), including Long Run Emphasis (LRE) and Run Length Nonuniformity (RLN) which also could be used to assess the homogeneity of the tumor^36^.

In our results, the radiomics scores of T2WI and CT are independent risk predictors to PNI-positive rectal cancer, which demonstrated the significant discrimination in the training dataset, and were approved in the testing dataset. CT score showed the best discrimination and calibration performance of the unimodality radiomics scores (training dataset, AUC = 0.874; testing dataset, AUC = 0.821). Our results confirmed that the published research revealed that integrated CT score and CEA level showed good calibration and discrimination performance (training dataset, AUC = 0.817; testing dataset, AUC = 0.803)^27^. Our research also highlights the role of T2WI score for the prediction of PNI status in rectal cancer. This result is in keeping with the published paper that the imaging biomarkers from MRI can be used to predict the biologic aggressiveness^37^. Following the present results, there was no statistical difference in the discrimination ability between the T2WI score and CT score (Supplementary Information 1: Table S2 and Table S3). Our research displayed a particular application of multi-modality radiomics in the interpretation of rectal cancer and gives a hypothesis that every modality radiomics feature provides different and independent information^28,38^. The concise score showed the best discrimination and calibration performance among the multi-modality radiomics scores that included T2WI score and CT score which were independent risk variables in the multivariate analysis. The discrimination ability of concise score is comparable in the training and testing datasets implies that radiomics score was robust for prediction and could be applied in the testing dataset. This is consistent with the result of clinical decision curve which showed the concise score and the integrated score have similar clinical application value, both of which are higher than MR score. Basing on the need for individual medical treatment the concise score was converted to the nomogram. It could generate an individual predictive probability of PNI-positive for each patient preoperatively. This easy-to-use system could help physicians and patients perform a preoperative prediction of PNI status and individualize the clinical management.

Our study found DWI score didn’t have additional predictive value to estimate PNI status in rectal cancer, while DWI is an important preoperative examination in rectal cancer^39,40^. We guessed several possible explanations for this phenomenon. First, lower spatial contrast resolution of DWI contrasting with CT images and T2WI affects the accuracy and robustness in the tumor segmentation. Our results revealed that 54% DWI radiomics features were eliminated because of low inter-/intra-observer reproducibility which is consistent with the published researches, only a small part of radiomics features from DWI remained independent and reproducible^41–43^. Second, portal venous phase CE-CT represents the enhancement of the tumor and therefore extracted quantitative features relating macroscopic blood flow^44^. In our results, twelve CT radiomics features were retained in the feature selection, even though more CT radiomics features were eliminated by ICCs than DWI radiomics features. This reason may be that PNI-positive rectal cancer is more likely to develop perivascular infiltration^3^. Furthermore, artifact-inducing influence can’t be neglected, and motion artifact is one of the most important causes limit DWI image quality^45,46^.

Our findings may be somewhat interpreted by some limitations. First, this is a retrospective study that excluded a lot of patients who didn’t perform preoperative rectal MR and CE-CT within 2 weeks, and thus there may exist potential selection bias in the study dataset. Second, Patients included in this study from a single-center in China without prognostic information, which restricts the predictive value and generalizability of these findings. In further research, a multi-center validation dataset should be included to evaluate the real benefit in this stratification and verify the robustness of the multi-modality Radiomics model. Third, there is no image pre-processing before the segmentation and radiomics feature extraction. Recent studies only indicated that signal normalization and voxel size resampling could decrease radiomics features’ variability. In the future, we will discuss how to improve the robustness of the model through image normalization of different devices and parameters^47,48^.

In conclusion, this research revealed the strong correlation of multi-modality radiomics features to the PNI status in rectal cancer. The multi-modality radiomics score may provide a valuable reference for clinicians in the individual clinical decision system.

## Methods

### Patients

This retrospective study was approved by the ethics committee of The First Hospital of Jilin University. The informed consent requirement was waived (IRB No. 19K060-001) and all procedures carried out were consistent with the ethical principles such as: International Health Organization good clinical practice (ICH-GCP) (showed in Supplementary Information 2 and 3). We reviewed the medical records of patients who underwent surgical resection for rectal cancer from June 2016 to October 2018. The inclusion criteria were as fellow: (I) patients who underwent surgery were diagnosed rectal cancer pathologically; (II) both rectal MRI and contrast-enhanced CT (CE-CT) were performed within 2 weeks before surgery; (III) PNI status was assessed by histopathological examination. The exclusion criteria were as follows: (I) lack of thick slices in the venous phase CT (n = 47); (II) patient underwent anti-tumor treatments before image examination (n = 33); (III) poor image quality (n = 7). The flowchart of the patient enrollment process is shown in Supplementary Information 1: Figure S5. Finally, we enrolled 94 consecutive patients, 70 (74.46%) men, and 24 (25.54%) women, with a median age of 60.5 years (interquartile range 50–68 years). These patients were randomly divided into training and testing datasets at a ratio of 7:3. Clinicopathologic data of eligible patients, including age, gender, carcinoembryonic antigen (CEA) and cancer antigen 19-9 (CA19-9), were derived from the medical records.

### Histopathology evaluation and perineural invasion status analysis

Histopathology results were obtained from the pathological information system (PIS). The rectal cancer staging and PNI status were evaluated based on the hematoxylin and eosin slides of the resected specimens according to the 8th AJCC staging system^49^. When the nervous structures and nerve sheaths were involved or more than 1/3 perineural space occupied by rectal tumor cells were defined as PNI-positive rectal cancer, otherwise were defined as PNI-negative rectal cancer.

### MR and CT imaging acquisition

All rectal MR and CT examinations were performed at our institution.MR imaging was acquired by a 3.0-T scanner (Ingenia, Philips Medical Systems, Netherlands) by using a phase-array coil. The analytic protocols and parameters were as follows: (I) high-resolution axial T2-weighted image (T2WI) was performed using fast recovery fast spin echo, repetition time (TR) = 3500 ms, echo time (TE) = 100 ms, slice thickness = 3.0 mm, gap = 0.3 mm, matrix = 288 × 256, echo train length = 24, and field of view (FOV) = 18 cm × 18 cm; (II) Diffusion-weighted imaging (DWI) was performed on b value = 1000 s/mm^2^, TR = 2800 ms, TE = 70 ms, slice thickness = 4.0 mm, gap = 1.0 mm, matrix = 256 × 256, and FOV = 34 cm × 34 cm. CT imaging was acquired by one of these two CT scanners (256-detector row, Brilliance, Philips Medical Systems, Netherlands; 64 slices dual source, Definition, Siemens Medical Systems). The protocol parameters of these two scanners were as follows: tube current = 250 mA; tube voltage = 120 kV; slice thickness = 5 mm; rotation time = 0.5- or 0.4-s; detector collimation = 8 × 2.5 mm or 64 × 0.625 mm; matrix = 512 × 512; FOV = 350 × 350 mm. The iodinated contrast (Ultravist 370, Bayer Schering Pharma, Berlin, Germany) was injected at a rate of 2.0–3.0 mL/s after a plain scan. The arterial and portal venous phase imaging were obtained at 25 s and 60 s after injecting. The final dose of iodinated contrast is 1.5 ml/kg.

### Imaging analysis and segmentation

MR and CT imaging were derived from the picture archiving and communication system (PACS, Agfa, Belgium) in the format of digital imaging and communications in medicine (DICOM). Two radiologists (Reader 1 and reader 2, respectively with 4 years’ and 10 years’ experience in rectal imaging) reviewed the MR and CT imaging of all included patients. Blinded to the histopathologic results, the radiologists had the primary tumor location and TNM staging according to the 8th AJCC staging system^50^. The thickness and length were respectively measured in the largest axial and sagittal plane. After daily readout sessions, consensus results were reached for all the patients.

Two radiologists (Reader 1 and reader 3, respectively with 3 years’ and 10 years’ experience in rectal imaging) manually segmented primary tumors using an open-source software package (ITK-SNAP version 3.4.0, http://www.itksnap.org) for each tumor slice in the modality of T2WI, DWI and portal venous phase of CE-CT. Either radiologist was blinded to the clinicopathological results. The volumes of interest (VOIs) segmented were defined as follows: (I) high-resolution T2WI images: manually drawn along the contour of the primary tumor lesion, which is slightly high signal; (II) DWI (b = 1000): manually drawn along the contour of the primary tumor lesion, which is high signal; (III) Portal venous phase of CE-CT: manually drawn along the primary tumor region enhanced heterogeneously which is could be differentiated from normal bowel structure. All VOIs were segmented on each primary tumor slice containing the circumambient chords and burrs, excluding the fluid in the intestinal lumen.

### Radiomics features extraction and selection

All VOIs in the modality of T2WI, DWI, portal venous phase of CE-CT were loaded to the A.K. software (Artificial Intelligence Kit, AK, version V3.0.0.R, GE Healthcare, China). 396 radiomics features (including 42 first-order statistics features, 345 high order statistics features, 9 Morphological Features) were extracted from every primary lesion VOI. Extraction and standardization of radiomics features was provided in the Supplementary Information 1: Figure S6.

30 VOIs were randomly selected to assess the inter-/intra-observer reproducibility of the Radiomics features extraction from each modality. Reader 1 is one of the two radiologists who performed tumor segmentation repeated this procedure after 2 weeks. Inter- and intra-class correlation coefficients (ICCs) were used to assess the reproducibility between the features which were extracted by reader 1 twice, as well as the features extracted by the two radiologists in the first-time. To ensure the robustness of the radiomics model, the features with ICCs more than 0.70 for both inter-observer and intra-observer were selected for subsequent analysis^50^. The mRMR algorithm and the LASSO algorithm were conducted to select the features with the best correlation to PNI status in the training dataset.

### Radiomics scores building and evaluation

The radiomics score was built via a linear combination of the selected features weighted by their respective coefficients based on the single modality alone. The radiomics score of T2WI, DWI, portal venous phase of CE-CT respectively present as T2WI score, DWI score, and CT score. Univariate logistic regression analysis was conducted to determine the Odd Ratio of the radiomics scores. Then multivariate logistic regression analysis with a backward stepwise elimination method was used to combine the radiomics scores using the Akaike information criterion (AIC).

**Figure 5:**
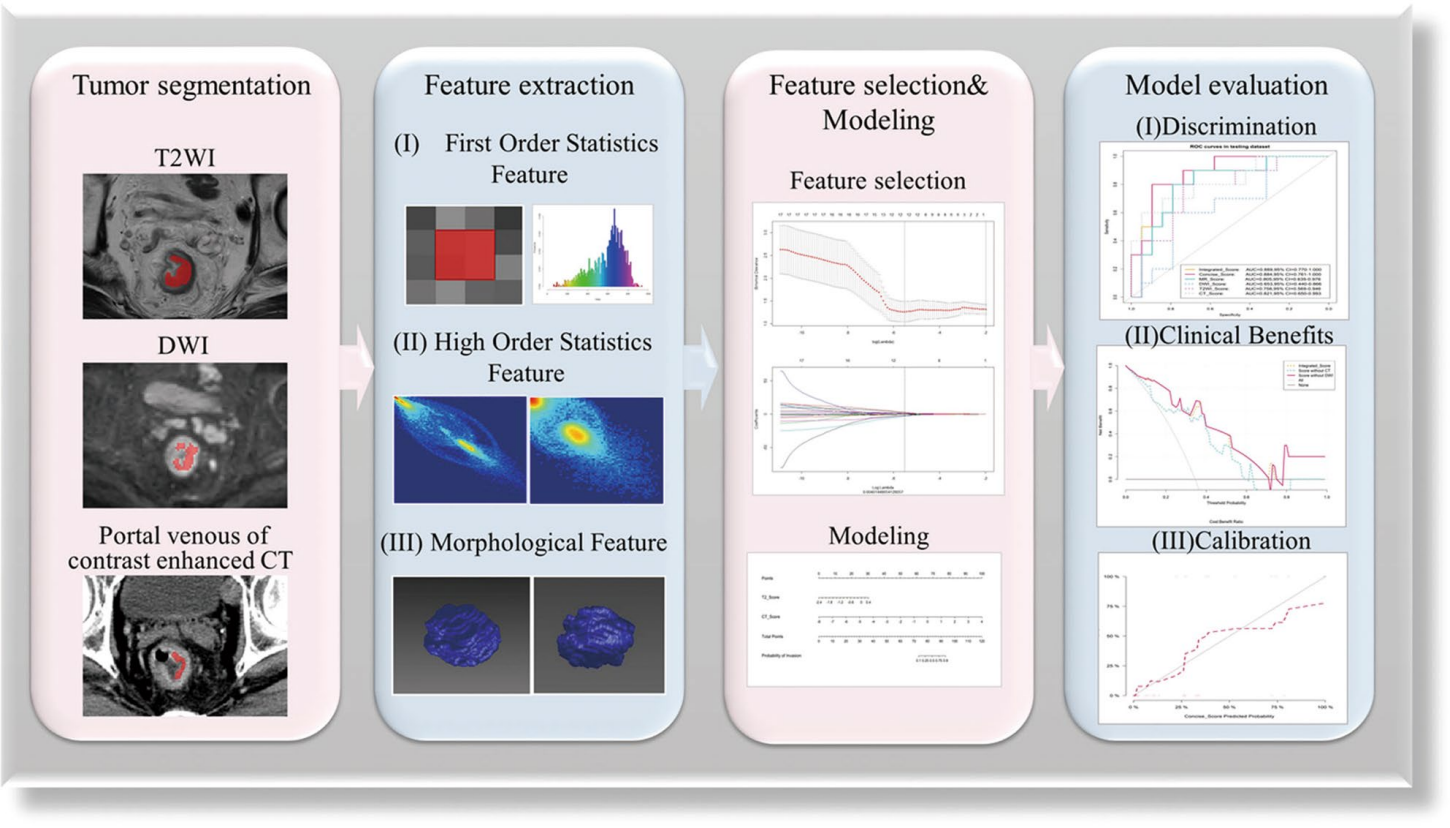
Flowchart describing the workflow of this study to construct and validate the radiomics models.

Accuracy, specificity, sensitivity, positive predictive value, negative predictive value, and the area under the receiver operating characteristic (ROC) curve (AUC) was used to evaluate the prediction performance of the models in both training and testing datasets. Decision curve analysis (DCA) was used to estimate the clinical value of the prediction models by contrasting the standardized net benefit at different risk threshold probabilities. The final model was selected which had a better discrimination ability and clinical benefit. The calibration curve depicted the consistency between the predicted probability value and the real value in the final model. A radiomics nomogram from the final model to ensure it is easy to use. All procedures building and validating radiomics models were shown in Fig. 5.

### Statistical analysis

All the statistical analyses were performed with R software (version 3.5.1, http://www.r-project.org). Age regarding as a continuous variable was expressed as mean ± standard deviation (SD). Sex was considered as a binary variable. CEA and CA19-9 were handled as binary variables cause the thresholds were set at 3.4 ng/ml and 22 ng/ml according to the clinical consensuses. The radiomics scores were expressed as a continuous variable and expressed as median (25% quantile, 75% quantile). The *t* test or Mann–Whitney *U* tests was performed to compare the continuous variables as appropriate. Fisher’s exact test or chi-squared test was applied to assess the distribution differences between the training and testing datasets in binary variables. Univariate and multivariate logistic regression analyses were respectively used to select the significant predictors from relevant clinicopathologic characteristics and radiomics scores to build prediction models. Delong tests were performed to assess the differences in AUC values between different radiomics models. Hosmer–Lemeshow goodness-of-fit test was applied in the calibration plot via bootstrapping resamples. In the statistical analysis P < 0.05 presented significant statistically. All packages used were listed in the supplementary Information 1: Table S1.

### Ethical statement

All the authors are responsible for guaranteeing the integrity and accuracy of the data collection and analysis process. This retrospective study was approved by the first hospital institutional ethics committee of Jilin University (IRB No. 19K060-001).

## Supplementary Information

Supplementary Information 1.

Supplementary Information 2.

Supplementary Information 3.

## Data Availability

The datasets generated during and/or analyzed during the current study are available from the corresponding authors on reasonable request.
