# Supplementary material for: Preoperative prediction of perineural invasion with multi-modality radiomics in rectal cancer
Source: Sci Rep. 2021 May 3;11:9429. doi: 10.1038/s41598-021-88831-2 (PMC8093213; doi:10.1038/s41598-021-88831-2)
Supplement: Supplementary file 2 — Supplementary Information 2. [file 41598_2021_88831_MOESM2_ESM.pdf]

吉林大学第一医院伦理委员会
临床试验与研究审批件

19K060-001

**项目名称**	肝癌、直肠癌区域化影像数据库建设		
**项目来源**	其他(结直肠癌标准化数据库的建立及大数据分析下新型诊疗模式探索)		
**专业名称**	放射线科	**主要研究者**	张惠茅
**审查类别**	初始审查	**审查方式**	会议审查
**投票结果**	同意10票，作必要修正后同意0票，作必要修正后重审0票，不同意0票，利益冲突退出0票		
**批准文件**	1.临床研究方案（版本号：1.0；版本日期：2019年03月28日）<br>2.知情同意书（版本号：1.0；版本日期：2019年04月07日）<br>3.记录试验数据的表格<br>4.主要研究者简历		
**审查意见**	同意		

根据ICH-GCP、卫计委《涉及人的生物医学研究伦理审查办法》（2016）、NMPA《药物临床试验质量管理规范（2003）》、《医疗器械临床试验规定（2004）》、《医疗器械监督管理条例》（2017）、《医疗器械临床试验质量管理规范》、WMA《赫尔辛基宣言》和CIOMS《人体生物医学研究国际道德指南》的伦理原则，经本伦理委员会审查，同意该临床试验在本中心开展。

请遵循GCP原则、遵循伦理委员会批准的方案开展临床研究，保护受试者的健康与权力。

研究过程中若变更主要研究者，对临床试验方案、知情同意书、招募材料等的任何修改，请申请人提交修正案审查申请。

发生严重不良事件，请申请人及时提交严重不良事件报告。

请申请人按照伦理委员会规定的年度/定期跟踪审查频率，在截止日期前1个月提交研究进展报告；当出现任何可能显著影响试验进行、或增加受试者危险的情况时，请申请人及时向伦理委员会提交书面报告。

研究纳入了不符合纳入标准或符合排除标准的受试者，符合中止试验规定而为未让受试者退出研究，给予错误治疗或剂量，给予方案禁止的合并用药等没有遵从方案开展的情况；或可能对受试者的权益/健康以及科学性造成不良影响等违背GCP原则的情况，请申办方/监查员/研究者提交违背方案报告。

申请人暂停/或提前终止临床研究，请及时提交暂停/终止研究报告。

完成临床研究，请申请人提交结题报告。

**年度/定期跟踪审查频率**	□3个月	□6个月	☑12个月
**有效期**	本批件有效期为一年，须在截止日期前1个月递交研究进展报告，经伦理委员会批准后方可继续进行。		
**(副)主任签字**	[illegible]		
**日期**	2019/4/27		

扫描全能王 创建
