# Supplementary material for: Preoperative prediction of perineural invasion with multi-modality radiomics in rectal cancer
Source: Sci Rep. 2021 May 3;11:9429. doi: 10.1038/s41598-021-88831-2 (PMC8093213; doi:10.1038/s41598-021-88831-2)
Supplement: Supplementary file 3 — Supplementary Information 3. [file 41598_2021_88831_MOESM3_ESM.docx]

**Ethics Committee of the First Hospital of Jilin University**

**Clinical Trials and Research Ethical Review Approval**

19K060-001

| Project Name | Construction of regional radiological imaging database for liver cancer and rectal cancer | | |
| --- | --- | --- | --- |
| Project Source | Establishment of a standardized database for colorectal cancer and exploration of new diagnosis and treatment model based on big data analysis | | |
| Department | Radiology department | The project leader | Huimao Zhang |
| Review Category | Primary Review | Review Method | Review conference |
| poll | 10 votes to agree, 0 votes to make necessary amendments for agreement, 0 votes to make necessary amendments for review again, 0 votes to disagree, 0 votes to exit for conflict of interest | | |
| Review  Documents | 1. Clinical study protocol   (version number:1.0; date:2019.03.28)   1. Informed consent   (version number:1.0; date:2019.03.28)  3. the table for recording experimental data  4. Curriculum Vitae of the project leader | | |
| In accordance with The ethical principles of the ICH-GCP, the health technology committee's “approach to the ethical review of biomedical research involving humans” (2016), “the NMPA's code for the quality management of pharmaceutical clinical trials (2003)”, “the medical device clinical trial regulations (2004)”, “the medical device regulatory regulation” (2017), “clinical trial quality management code for medical devices”, “declaration of Helsinki” of WMA and “international guidelines for human biomedical research” of CIOMS are reviewed by this ethics committee, and the clinical trial is approved to be carried out in this center.  Please conduct the clinical study in accordance with the GCP principles and the protocol approved by the ethics committee to protect the health and rights of the subjects.  If the principal investigator is changed during the study, and any modifications are made to the clinical trial protocol, informed consent, and recruitment materials, the applicant is requested to submit an application for amendment review.  If serious adverse events occur, please submit the report of serious adverse events in time.  Subjects who did not meet the inclusion criteria or exclusion criteria were included in the study; subjects who did not withdraw from the study despite meeting the test suspension requirements were given the wrong treatment or dose; In the event of a GCP violation that may adversely affect the rights/health and scientific nature of the subject, the sponsor/inspector/investigator is requested to submit a protocol violation report.  Applicants who suspend/terminate the clinical study should submit the study suspension/termination report in a timely manner.  After completing the clinical study, please submit the final report. | | | |
| Review Decision | Agreement | | |
| Track Review Frequency Annually/Periodically | □3 months □6 months ☑1 year | | |
| Period of Validity | This approval is valid for 1 year. A progress report shall be submitted 1 month before the deadline. It should be approved by the ethics committee before proceeding | | |
| Signature of (deputy) director |  | | |
| Date | 2019.06.27 | | |
