# Supplementary material for: Preoperative prediction of perineural invasion with multi-modality radiomics in rectal cancer
Source: Sci Rep. 2021 May 3;11:9429. doi: 10.1038/s41598-021-88831-2 (PMC8093213; doi:10.1038/s41598-021-88831-2)
Supplement: Supplementary file 1 — Supplementary Information 1. [file 41598_2021_88831_MOESM1_ESM.docx]

**Title Page**

**Title：**

Preoperative Prediction of Perineural Invasion with Multi-modality Radiomics in Rectal Cancer

**Author List:**

Yu Guo^1^, Quan Wang^2^, Yan Guo^3^, Yiying Zhang^1^, Yu Fu^1^*****, Huimao Zhang^1^*****

**Authors’ Affiliations:**

^1^ Department of Radiology, The First Hospital of Jilin University; Jilin Provincial Key Laboratory of Medical imaging & big data, Changchun, China

^2^ Department of Gastric and Colorectal Surgery, The First Hospital of Jilin University, Changchun, China

^3^ GE Healthcare, Shanghai, China

***** corresponding author E-mail: [fuyujdyy@163.com](mailto:fuyujdyy@163.com); [huimaozhanglinda@163.com](mailto:huimaozhanglinda@163.com)

**Figure S1. T2WI Radiomics features selection using the (LASSO) algorithm**

20 T2WI Radiomics features selection using the least absolute shrinkage and selection operator (LASSO) binary logistic regression algorithm. (a) Tuning parameter lambda selection in the LASSO algorithm used 5-fold cross-validation via minimum criteria. The dotted vertical lines were drawn at the optimal lambda’s value by using the minimum criteria of the standard error. lambda’s value is 0.051. (b) 20 T2WI radiomics features’ coefficient in the LASSO algorithm and the coefficient curve was produced against the log lambda. The dotted Vertical line was drawn at the value selected using 5-fold cross-validation, where optimal lambda resulted in 5 nonzero coefficients. (c) The corresponding 5 nonzero coefficients and respective selected T2WI radiomics features.

**Figure S2. DWI Radiomics features selection using the (LASSO) algorithm**

20 DWI Radiomics features selection using the least absolute shrinkage and selection operator (LASSO) binary logistic regression algorithm. (a) Tuning parameter lambda selection in the LASSO algorithm used 5-fold cross-validation via minimum criteria. The dotted vertical lines were drawn at the optimal lambda’s value by using the minimum criteria of the standard error. lambda’s value is 0.095. (b) 20 DWI radiomics features’ coefficient in the LASSO algorithm and the coefficient curve was produced against the log lambda. The dotted Vertical line was drawn at the value selected using 5-fold cross-validation, where optimal lambda resulted in 1 nonzero coefficient. (c) The corresponding 1 nonzero coefficient and respective selected DWI radiomics features.

**Figure S3.** **Portal venous phase** **CE-CT Radiomics features selection using the (LASSO) algorithm**

20 portal venous phase CE-CT Radiomics features selection using the least absolute shrinkage and selection operator (LASSO) binary logistic regression algorithm. (a) Tuning parameter lambda selection in the LASSO algorithm used 5-fold cross-validation via minimum criteria. The dotted vertical lines were drawn at the optimal lambda’s value by using the minimum criteria of the standard error. lambda’s value is 0.004. (b) 20 portal venous phase CE-CT radiomics features’ coefficient in the LASSO algorithm and the coefficient curve was produced against the log lambda. The dotted Vertical line was drawn at the value selected using 5-fold cross-validation, where optimal lambda resulted in 12 nonzero coefficients. (c) The corresponding 12 nonzero coefficients and respective selected portal venous phase CE-CT radiomics features.

**Figure S4. Bar chart of concise score in training and testing dataset.**

(a)Training dataset;(b) Testing dataset. Up bars refer to the predicted positive perineural invasion; Down bars refer to the predicted negative perineural invasion. Green bars represent perineural invasion status is positive; Red bars represent perineural invasion status is negative.

**Figure S5. Flowchart of the patient enrollment process.**

**Figure S6. The category of the extracted radiomics features**

After radiomics features extraction, a standardization was performed to improve robustness and generalization ability of radiomics model. The formula for standardizing imaging parameters is:

$$standardization=\frac{fi-u}{\mathrm{std}}$$

fi，radiomics feature value; u: average radiomics feature value; std, standard deviation of radiomics feature value.

**Supplementary TableS1.** All packages in the procedure of statistical analysis

| Procedure of statistical analysis | Packages |
| --- | --- |
| LASSO regression analysis | “glmnet” package |
| mRMR analysis | “mRMRe” package |
| Univariate logistic regression | “rms” package |
| Multivariate logistic regression | “rms” package |
| Nomogram Construction | “rms” package |
| calibration plot | “rms” package |
| ROC curves | “pROC” package |
| Decision curve analysis | “dca. R” package |

All the reported significant statistical level was all two-sided and the statistical significance set at 0.05.

| **Supplementary TableS2**. The discrimination performance difference of every radiomics signatures and derived radiomics models in the training dataset. | | | | | | |
| --- | --- | --- | --- | --- | --- | --- |
| P value | T2 | DWI | CT | MR_Score | Int_Score | Concise_Score |
| T2 | - | - | - | - | - | - |
| DWI | 0.138 | - | - | - | - | - |
| CT | 0.233 | 0.003 | - | - | - | - |
| MR_Score | 0.398 | 0.040 | 0.296 | - | - | - |
| Int_Score | 0.032 | ＜0.001 | 0.404 | 0.042 | - | - |
| Concise_Score | 0.027 | ＜0.001 | 0.354 | 0.035 | 0.382 | - |

P value was derived from Delong test between radiomics signatures and derived radiomics models in the training dataset.

| **Supplementary TableS3.** The discrimination performance difference of every radiomics signatures and derived radiomics models in the testing dataset. | | | | | | |
| --- | --- | --- | --- | --- | --- | --- |
| P value | T2 | DWI | CT | MR_Score | Int_Score | Concise_Score |
| T2 | - | - | - | - | - | - |
| DWI | 0.438 | - | - | - | - | - |
| CT | 0.654 | 0.039 | - | - | - | - |
| MR_Score | 0.148 | 0.178 | 0.898 | - | - | - |
| Int_Score | 0.102 | 0.013 | 0.374 | 0.170 | - | - |
| Concise_Score | 0.123 | 0.015 | 0.416 | 0.203 | 0.480 | - |

P value was derived from Delong test between radiomics signatures and derived radiomics models in the testing dataset.
